# Inducers of Senescence, Toxic Compounds, and Senolytics: The Multiple Faces of Nrf2-Activating Phytochemicals in Cancer Adjuvant Therapy

**DOI:** 10.1155/2018/4159013

**Published:** 2018-02-12

**Authors:** Marco Malavolta, Massimo Bracci, Lory Santarelli, Md Abu Sayeed, Elisa Pierpaoli, Robertina Giacconi, Laura Costarelli, Francesco Piacenza, Andrea Basso, Maurizio Cardelli, Mauro Provinciali

**Affiliations:** ^1^Advanced Technology Center for Aging Research, Scientific Technological Area, Italian National Institute of Health and Science on Aging (INRCA), Ancona 60121, Italy; ^2^Department of Clinical and Molecular Sciences, Polytechnic University of Marche, Ancona 60126, Italy

## Abstract

The reactivation of senescence in cancer and the subsequent clearance of senescent cells are suggested as therapeutic intervention in the eradication of cancer. Several natural compounds that activate Nrf2 (nuclear factor erythroid-derived 2-related factor 2) pathway, which is involved in complex cytoprotective responses, have been paradoxically shown to induce cell death or senescence in cancer. Promoting the cytoprotective Nrf2 pathway may be desirable for chemoprevention, but it might be detrimental in later stages and advanced cancers. However, senolytic activity shown by some Nrf2-activating compounds could be used to target senescent cancer cells (particularly in aged immune-depressed organisms) that escape immunosurveillance. We herein describe in vitro and in vivo effects of fifteen Nrf2-interacting natural compounds (tocotrienols, curcumin, epigallocatechin gallate, quercetin, genistein, resveratrol, silybin, phenethyl isothiocyanate, sulforaphane, triptolide, allicin, berberine, piperlongumine, fisetin, and phloretin) on cellular senescence and discuss their use in adjuvant cancer therapy. In light of available literature, it can be concluded that the meaning and the potential of adjuvant therapy with natural compounds in humans remain unclear, also taking into account the existence of few clinical trials mostly characterized by uncertain results. Further studies are needed to investigate the therapeutic potential of those compounds that display senolytic activity.

## 1. Introduction

Cellular senescence (CS) is a biological response to a variety of stresses that results in persistent growth arrest with a distinct morphological and biochemical phenotype [1–3]. It is currently considered a “barrier” to prevent malignant transformation and a potent anticancer mechanism as well as a hallmark of aging. Exploration of CS to drive towards antitumor adjuvant therapies by natural compounds is currently gaining increasing interest. Cancer cells can be forced to undergo senescence by natural compounds, with effects somewhat comparable to those obtained by genetic and epigenetic manipulations, anticancer drugs, and irradiation [4]. These effects have been shown after sustained exposure *in vitro* to a wide range of different substances that are also paradoxically used to obtain cytoprotective and chemopreventive adaptive responses in normal cells [5, 6]. Interestingly, most of these cytoprotective activities are likely to be mediated by Nrf2 (nuclear factor erythroid-derived 2 related factor 2) stress-responsive signaling [7–9]. Examples of these natural bioactive compounds include mostly phenols like curcumin, epigallocatechin gallate (EGCG), fisetin, genistein, phloretin, quercetin, resveratrol, and silybin as well as other classes of compounds such as organosulfur compounds [i.e., allicin, phenethyl isothiocyanate (PEITC), and sulforaphane], metyl-tocols [i.e., tocotrienols], alkaloids (i.e., berberine, piperlongumine), and terpenoids (i.e., triptolide) [9–12]. Although, in certain cases, these compounds can specifically interact with the altered pathways of cancer cells [5]; the structural and physical differences of these compounds suggest that their ability to activate the antioxidant response elements (AREs) of many cytoprotective genes through the cytoplasmic oxidative stress system, Nrf2-Keap1 (Kelch-like ECH-associated protein 1), is perhaps a common mechanism of action. Considering that cancers with high Nrf2 levels are associated with poor prognosis because of radio and chemoresistance and aggressive proliferation, activating Nrf2 pathway is considered protective in the early stages of tumorigenesis but detrimental in the later stages [13]. Hence, it can be found a paradox on how Nrf2-activating compounds can be proposed to induce senescence in cancer cells and, eventually, as a tool for adjuvant therapy. Interestingly, it is becoming evident that some effects of Nrf2-Keap1 pathway may be mediated through crosstalk with additional pathways (i.e., the aryl hydrocarbon receptor (AhR) pathway) affecting aspects of cell fate that provide a multitiered, integrated response to chemical stresses [14] which, in turn, could eventually culminate in a senescent response. This could be promoted by defective pathways of cancer cells or by excess amounts of the bioactive compounds. Indeed, most of the prosenescence effects shown *in vitro* are obtained with relatively high concentrations of the bioactive compounds (micromolar ranges) that are likely to not be translated *in vivo* (usually nanomolar ranges) due to potential toxicity to healthy cells, unless the compound can be specifically targeted to cancer cells. Interestingly, selective accumulation of natural compounds (i.e. T3s) in cancer tissues has been reported [15] and would deserve appropriate investigation for the future development of adjuvant supplements in cancer therapy. The possibility to induce senescence in tumors with lower drug doses, especially if administered chronically, may potentially limit treatment-related toxic side effects. However, even in the cases where a sufficient degree of selectivity has been demonstrated, senescence escape systems of cancer cells [16] may hamper the efficacy and thus the clinical applications of these compounds. In addition, it is an emerging concept that immune responses against senescent cells are crucial to restrict disease progression in cancer pathologies [17]. Treatments aimed at inducing senescence in cancer are likely to fail in the complete clearance of senescent cancer cells if not supported by a proper senescence immune surveillance response [16]. Senescent cancer cells might later be able to revert their senescent phenotype [18] or promote new cancers in their microenvironment in the case of ineffective clearance mechanisms [19, 20]. In this case, the interaction of natural bioactive compounds with the senescent-associated secretory phenotype (SASP) might be crucial. The SASP may have positive or negative effects, depending on the context: it can cause local and potentially systemic inflammation, disrupt tissue architecture, and stimulate growth and survival of nearby malignant cells [17], but it can be eventually important to promote immune clearance of senescent cells. Hence, the anti-inflammatory activity of natural compounds should also be carefully evaluated in this context. Additional challenges in this field include the proper characterization of CS [21], a clear understanding of the role of senescent cells in physiological and pathological conditions [2] and the huge heterogeneity of CS models [22, 23] which, in turn, might make it difficult to compare the effects and understand the proper area of application of promising natural bioactive compounds. In this review, we summarize the most relevant studies focused on induction of CS *in vitro* by selected bioactive compounds and discuss critical aspects related to their putative mechanisms of action and eventual translation *in vivo*.

## 2. Cellular Senescence and Senolytic Compounds

CS is usually defined as a status of growth arrest mediated by insensibility to mitogen stimuli, chromatin and secretome changes, and upregulation of particular tumor suppressor pathways [2, 24, 25]. CS induction may occur by a variety of cell-intrinsic and cell-extrinsic stresses, including DNA damage, oxidative stress, critical telomere shortening and damage, chronic mitogenic signaling, oncogene activation and inactivation, loss of tumor suppressors, nucleolar stress, and epigenetic changes [25]. There is no unique and definitive marker to define the senescent status of a cell, and not all senescent cells show the same features. Hence, characterization of CS can be performed by assessing multiple markers such as an enlarged morphology, the activation of p53-p21 and/or p16-Rb tumor suppressor pathways, the presence of persistent DNA damage response (DDR), an increase in CS-associated beta-galactosidase (SA-*β*-Gal) activity, and the appearance of senescent-associated distension of satellites and telomere-associated DNA damage foci. In replicative senescence, critically shortened telomeres activate DDR and subsequent stabilization of p53 while oxidative stress and oncogene-induced senescence may work mainly through the activation of both p53 and p16 pathways [2]. Epigenetic derepression of CDKN2A (cyclin-dependent kinase inhibitor 2A) locus, which encode for both p16 and p14, is another trigger for senescence associated with aging and is responsible of the increased expression of p16 in aged tissues [26] and one of the most prominent indicators of the presence of senescent cells in aged tissues [27]. A common mediator of CS is the inhibition of Rb phosphorylation, which results in the inactivation of the E2F transcription factor, and its target genes involved in cell cycle progression [28]. The activation of growth pathways, via the mTOR (mammalian target of rapamycin), and the autophagic response appear as additional important players in establishing CS [29]. Senescent cells additionally display an increase in metabolic activity and, frequently, develop the SASP, which includes several proteins involved in inflammation processes, proteases, hemostatic, and growth factors [30].

Studies of human tissues and cancer-prone mice argue strongly that CS is one of the most important processes to suppress cancer *in vivo* [24], but the SASP produced by senescent cells can induce deleterious effects in the microenvironment by damaging neighboring cells, thus facilitating tumor development and aggressiveness [31], mediating paracrine transmission of CS [32], and promoting age-related dysfunctions [25].

An important physiological function of the SASP is to promote clearance of senescent cells by the immune system (a process named senescence immunosurveillance). However, age-related immunodeficiency or the production of a less proinflammatory SASP by senescent cells accumulated in aged-tissues could hamper senescence immunosurveillance [25].

A relevant feature of some, but not all [33], senescent cells is their long-term survival and resistance to apoptosis [34], which likely contributes to their persistence and the respective deleterious consequences in aged tissues. A direct demonstration that senescent cells can drive age-related pathologies has been originally provided with the development of a transgenic mouse model, in which p16-expressing cells can be specifically eliminated upon drug treatment, with consequent prevention, delay, or attenuation of some age-related disorders [35]. This study prompted the birth of a field of research aimed to identify antiapoptotic mechanisms occurring in senescent cells and the relative compounds that are able to break this resistance to cell death with a high selectivity for senescent cells. The development of this area of research has been so fast that research around compounds able to selectively induce death in senescent cells (named senolytic drugs) represents now one of the most fruitful area of investigation [36, 37]. Preclinical studies have reported that senolytic compounds can improve cardiac function [38] in old mice, recover vascular function and decrease vascular calcification in atherosclerosis mice [39], and improve pulmonary function and physical health in mouse models of fibrotic pulmonary disease [40], as well as achieve partial rejuvenation in several tissues of progeroid mice [38, 41]. Besides, these are only part of the results achieved in age-related chronic conditions and others are expected to be shown soon. Importantly, senescent cells accumulate in mice treated with chemotherapy, causing a range of defects and promoting tumor recurrence [42]. Hence, it is not surprising that senolytic compounds have been proven to delay tumor recurrence and metastasis in mouse cancer models after chemotherapy [42] as well as to ameliorate side effects associated with the therapy [43]. However, senolytic compounds appear to be cell type restricted as a consequence of the heterogeneity of senescent cells and their different antiapoptotic pathways. The most important antiapoptotic pathways identified in senescent cells include the B-cell lymphoma 2 (BCL-2)/B-cell lymphoma-extra large (Bcl-xL), the PI3k*δ*/AKT, the p53/p21, the ephrins, the HIF-1*α*, the HSP-90, and various metabolic pathways [37]. These pathways may be differentially activated depending on the type of senescent cells (e.g., endothelial cells or fibroblasts) and the species of the donor (e.g., human or mice); thus, each senolytic compound displays its activity in some but not all types of senescent cells. In certain cases, the combination of two compounds is effective as senolytic in a wider range of cell type than the single compounds. This is the case of the combination of quercetin with dasatinib, which is effective in several models of senescent cells (endothelial, preadipocytes, and fibroblasts), whereas quercetin is only effective in radiation-induced endothelial cells and dasatinib in senescent preadipocytes [38].

**Figure 1:**
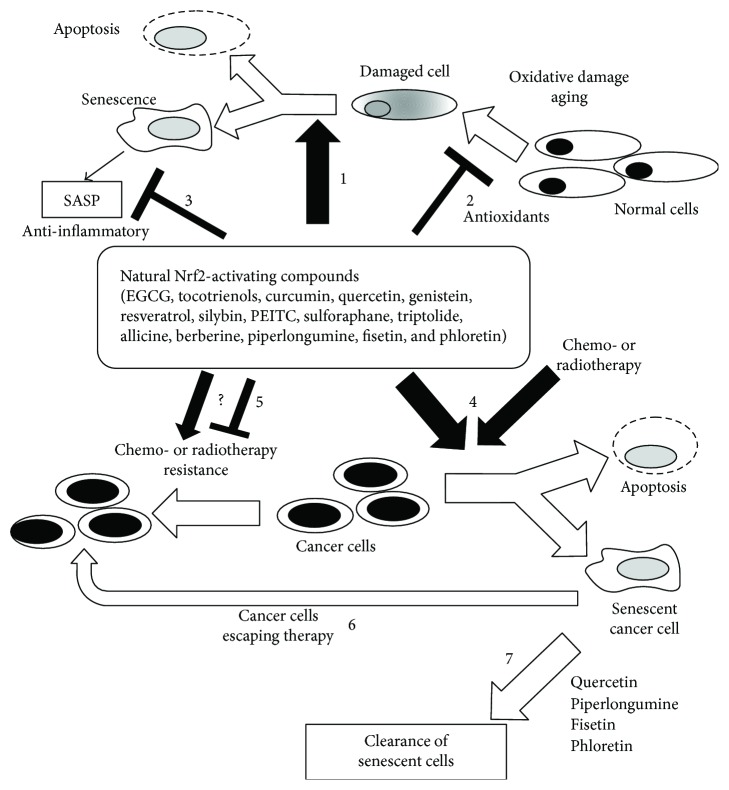
Potential effects and concerns of selected natural compounds as adjuvant in cancer therapy. Based on experiments “in vitro,” epigallocatechin gallate (EGCG), tocotrienols, curcumin, quercetin, genistein, resveratrol, silybin, phenyl isothiocyanate (PEITC), sulforaphane, triptolide, allicin, berberine, piperlongumine, fisetin, phloretin might be useful in prevention and therapy of cancer. Gero- and cancer preventive activity include (1) induction senescence or apoptosis in normal damaged and potentially precancerous cells, (2) protection of normal cells by damage via modulation of antioxidant/cytoprotective pathways, and (3) anti-inflammatory activity that might reduce negative effects of the senescence-associated secretory phenotype (SASP) produced by senescent cells. In cancer therapy, natural bioactive compound might help (4) to induce apoptosis and senescence in cancer cells thus helping to reduce dosage of chemo- and radiotherapy while keeping efficacy. The major concern regards the possibility that these compounds might act as cytoprotective in some cancer cells (as in normal cells), thus aggravating the problem of resistance of cancer to therapy (5). However, failure to clearance senescent cells (6), as it might occur in immune-compromised subjects, might represent a serious challenge for these applications. Inclusion of additional strategies (7) with other natural compounds (i.e., phloretin, fisetin, piperlongumine, and quercetin) able to induce selective death of senescent cells should be evaluated in future preclinical studies to reduce relapses and side effects of chemo- or radiotherapy.

Up to date, a small number of natural compounds have been shown to display senolytic activity, but it is likely that this is the tip of an iceberg that will be exposed in the coming years. These include quercetin [38], fisetin [44], phloretin [45], and piperlongumine [46], and there are preliminary indications that tocotrienols [47] and, eventually, cannabinoids [6] may also display senolytic activity in particular models of cellular senescence. Paradoxically, and very similar to the activity of senescence inducers described in the previous section, all these compounds can be accumulated by their potential to induce Nrf2 cellular response, which has a well-demonstrated cytoprotective and antiapoptotic effects. Indeed, the Nrf2/Keap1 pathway is activated by quercetin [48], fisetin [49], piperlongumine [50], and phloretin [51] as well as by different cannabinoids [52], tocotrienols [53], and a multitude of anticarcinogenic natural compounds that were found to cause cancer cell apoptosis or senescence under certain conditions (Figure 1).

The molecular mechanisms explaining how the Nrf2/Keap1 pathway is modulated during apoptosis and senescence are currently largely unknown, and this gap of knowledge may contribute to hamper the clinical translation of adjuvant therapies based on Nrf2-activating compounds.

## 3. The Nrf2-Keap1 Pathway

The Nrf2-Keap1 pathway is a key controller of cellular response to stress caused by reactive oxygen species (ROS) [13]. The Nrf2 antioxidant response is mediated by the activation of ARE/electrophile responsive element (ARE) in the regulatory region of target genes. Molecular details of this signaling pathway and its dysregulation in cancer have emerged over the last 10 years and are extensively reviewed elsewhere [13, 14, 54]. Oxidative signals induce changes in the sulfhydryl groups of Keap1, thus promoting Nrf2 dissociation from Keap1, Nrf2 nuclear translocation, and stimulate mRNA expression of Nrf2-targeted downstream genes. Genomic analyses indicate that gene families affected by Nrf2 display a multitude of responses with a defensive role against cellular senescence including detoxification, antioxidant, damage repair, and inhibition of inflammation. This response involves more than 200 antioxidant and protective genes that constitute the so-called phase II response. Among these enzymes, we can mention *γ*-glutamylcysteine ligase (*γ*-GCL), glutathione peroxidase (GPx), heme oxygenase 1 (HO-1), superoxide dismutase (SOD), glutathione S-transferase (GST), and NADPH-quinone oxidoreductase (NQO1) which have been frequently studied in the context of a protective response against cell death or senescence [55]. Hence, it is not surprising that a body of evidence supports the role of Nrf2 in mediating protection against stress-induced senescence [55–58]. Several studies have identified inactivating mutations in Keap1, leading to an increase in Nrf2 function, in human cancers [59–61]. Hence, inducing the Nrf2-regulated cytoprotective response could provide a selective advantage to tumor cells, raising the question of whether it is hazardous to elicit these changes in the context of interventions for cancer.

The role of Nrf2 in cellular senescence has been poorly studied. It is known by other models (cell lines and cancer cells) that Nrf2 upregulates most of the antiapoptotic mechanisms that have been shown to be repressed by senolytics, including natural compounds. In particular, HIF-1 alpha signaling is augmented by induction of the Nrf2 pathway, as demonstrated in hypoxia models [62]. Moreover, Nrf2 protein upregulates the antiapoptotic protein Bcl-2 [63] and interacts with p21, which promotes the activation of the antioxidant response mediated by Nrf2 [64]. Hence, it would have been expected that Nrf2 is upregulated in cellular senescence. In contrast with this rationale, Nrf2 has a declined function in senescence of human fibroblasts [65], whereas its silencing leads to premature senescence [65, 66]. Moreover, it appears to be downregulated in oncogene-induced senescence of transformed cell lines (in which senescence can be triggered by MEK activation) and upregulated when senescence is bypassed (GEO Accession: GDS1637, Profile GDS1637/201146_at) [67]. In contrast with the premature senescence induced by Nrf2, others have reported that deletion of Nrf2 in mouse embryonic fibroblasts is associated with immortalization [68]. Interestingly, these immortalized cells display a positive staining for SA-*β*-Gal, thus suggesting that deficiency in proteosomal degradation induced by Nrf2 deletion could be compensated, at least in part, by induction of lysosomal hydrolytic enzymes [68] that are similarly activated in several models of cellular senescence.

In the next chapters, we describe the most studied Nrf2-inducing natural compounds that have been shown to act *in vitro* as toxic compounds in cancer cells and that can be used as senolytics or senescence inducers (or both). A particular focus on the doses used in the experiments and a short description of their eventual use in clinical trials is also provided as schematically represented in Figure 1.

## 4. Nrf2-Activating Phytochemicals: Senescence Inducers, Senolytics, or Toxic Compounds for Cancer Cells

### 4.1. Tocotrienols

Tocotrienols (T3s), members of the vitamin E family, are naturally occurring compounds composed of four different isomers: alpha (*α*), beta (*β*), gamma (*γ*), and delta (*δ*). These compounds are present in barley oil, coconut oil, corn oil, palm oil, rice bran oil, wheat germ, and annatto [69]. Cell culture studies suggest that T3s affects numerous pathways linked with tumorigenesis, including Nrf2 pathway [47, 53].

#### 4.1.1. Tocotrienols as Toxic Compounds in Cancer

In the last years, T3s have been of increasing interest due to the discovery of their anticancer effects, not generally evident with tocopherol-rich vitamin E preparations [70]. Among the four isoforms of T3s, *γ* and *δ* are those which have proven greater effectiveness in countering the proliferation of tumor cells. T3s can induce apoptosis in various types of mammary cancer cells by acting on mitochondrial or death receptor-mediated pathways [71, 72].

Further anticancer mechanisms of T3s including downregulation of mitogenic signal/survival factors and induction of paraptosis-like death have also been described in different cellular models.

#### 4.1.2. Tocotrienols as Senescence Inducers

T3s have been additionally shown to induce cell cycle arrest and senescence-like phenotype in various cancer cells *in vitro*. Genes involved in cell cycle control, such as p21, p27, and p53, may represent the downstream effectors of T3s that affect the balance between signals that drive the cell into senescence or to death. In malignant mouse +SA mammary epithelial cells, 4 *μ*M *γ*-tocotrienol significantly inhibited cell proliferation which was associated with reduction in cell cycle progression from G_1_ to S, as evidenced by increased p27 level, and a corresponding decrease in cyclin D1, CDK (cyclin-dependent kinase) 2, CDK4, CDK6, and phospho-Rb levels [73]. Similar results have been shown in HER-2 (human epidermal growth factor receptor 2) overexpressing cell lines with the upregulation of p53, p21, and p16 induced by mixtures of *γ*-T3s and *δ*-T3s [72]. Interestingly, oral administration of 100 mg/kg annatto-T3 delayed the spontaneous onset of mammary tumor and reduced tumor number and size through enhancing in situ both apoptosis and senescence markers in a HER2/neu breast cancer mouse model [15], thus showing that the results obtained *in vitro* can be translated *in vivo*. In this mouse model, T3s have been shown to specifically accumulate in cancer tissues of HER2/neu mice at a very high rate than observed in normal tissues.

T3s (10–20 *μ*M) have also been shown to inhibit telomerase by affecting hTERT (human telomerase reverse transcriptase) and c-Myc expression through PKC (protein kinase C) activity suppression in human colorectal adenocarcinoma cell lines [74]. By the way, c-Myc is known to induce epigenetic changes leading to transcriptional activation of genes that suppress key drivers of CS. The involvement of PKC, whose isoforms are known to selectively mediate certain malignant phenotype including HER2-positive breast tumors [75], in the mechanisms of action of T3s might also contribute to explain why T3s can induce opposite effects (antisenescence) in normal human fibroblasts [76]. Another upstream target of T3s that could mediate senescent-like response or apoptosis in breast cancer cells is ERs (estrogen receptors) [77]. T3s display high affinity for ER*β* and increase its translocation into the nucleus which, in turn, activates the expression of estrogen-responsive genes [MIC-1 (macrophage inhibitory cytokine-1), EGR-1 (early growth response protein 1), and cathepsin D] involved in growth arrest, altered morphology, and apoptosis of ER*β* expressing breast cancer cells (MDA-MB-231 and MCF-7) [78]. Hence, the idea that these compounds might promote senescence in cancer cells while displaying antisenescence effects in normal cells sounds very promising in view of its potential clinical applications.

#### 4.1.3. Tocotrienols as Potential Senolytics

Senolytic activity has not been tested for T3s. However, some metabolic and apoptotic pathways affected by these compounds in cancer cells overlap with those of other compounds that have been shown to display senolytic activity, such as quercetin [47]. Moreover, T3s have been shown to display rejuvenating effects which might in the end represent the net results of a senolytic activity on senescent cells and a selective survival of a subpopulation of nonsenescent cells in the culture.

#### 4.1.4. Tocotrienols in Cancer Adjuvant Therapy

Despite the number of clinical trials conducted to examine the multifaceted health benefits of T3s [79, 80], very little is known about the efficacy of T3s as adjuvant supplements in cancer therapy. Pilot clinical trials on the synergistic effect of T3s and chemotherapy have been mainly addressed to test safety without any clear advantage for survival or other clinical endpoints [81, 82]. However, measurements of T3s in malign and benign adipose breast tissues of a Malaysian population found that total T3s levels were lower in the malignant tissues compared to the benign ones [83]. These data reinforce the idea that T3s may provide some kind of protection against breast cancer but the circumstances and the modality of intervention would require further studies.

### 4.2. Curcumin

Curcumin, a component of turmeric rhizome, is another example of Nrf2-activating compound [84, 85] that, in certain circumstances, acts as cytotoxic or prosenescence compound in cancer cells.

#### 4.2.1. Curcumin as Toxic Compound in Cancer

Curcumin affects various biochemical and molecular cascades involved in cancer by acting on a multitude of molecular targets including NF-*κ*B (nuclear factor kappa-light-chain-enhancer of activated B cells), Akt, MAPK (mitogen-activated protein kinases), p53, Nrf2, Notch-1, JAK (Janus kinase)/STAT (signal transducer and activator of transcription), *β*-catenin, and AMPK (5′ adenosine monophosphate-activated protein kinase) [86]. A direct inhibition of mTORC1 (mammalian target of rapamycin complex 1) signaling [87] and induction of autophagic cell death [88] have also been claimed to explain the cytotoxic effects of curcumin in various cancer cells. However, the same mechanisms can also be responsible for the reversion of senescence and appearance of proliferating cells in irradiated apoptosis-resistant cells [89].

#### 4.2.2. Curcumin as Senescence Inducer

Notwithstanding senescence-suppressive activity, there is substantial evidence that curcumin can induce senescence in different cancer models. This has been clearly shown in MCF-7 breast cancer cell line [90, 91], human colon cancer cells, [90, 92] and breast cancer-associated stromal fibroblasts [93]. Inhibition of telomerase activity, induction of p53, p21, and p16, and an increased autophagic response have been reported as the main mediators of this prosenescence activity of curcumin.

#### 4.2.3. Curcumin as Potential Senolytic

Curcumin has been recently tested for a potential senolytic activity in senescent fibroblasts from Ercc1−/− mice showing no effect on senescent cells [41]. However, this kind of activity would deserve further experiments in different type of cells.

#### 4.2.4. Curcumin in Cancer Adjuvant Therapy

The most likely explanation to this multitude of proposed mechanisms is that curcumin can display cell-specific effects, thus suggesting that adjuvant therapy with this compound could be most effective in certain type of cancer using appropriate delivery systems. Some promising effects have been observed in breast, prostate, lung, pancreatic, and colorectal cancer as well as in multiple myeloma [94]. Consistent with *in vitro* studies, curcumin administration has been shown to affect molecular targets involved in cancer. Presurgery curcumin administration in patients with colorectal cancer decreased serum TNF-alpha levels and increased cancer cell apoptosis, observed as enhanced p53 and Bcl-2, and decreased Bax expression in tumor tissues compared with control [95]. A decrease in NF-*κ*B and cyclooxygenase-2 (COX-2) expression and pSTAT3 activation was shown in peripheral blood mononuclear cells (PBMC) from patients with advanced pancreatic cancer receiving curcumin oral administration contemporary to gemcitabine-based chemotherapy [96]. Also, curcumin seems to be effective in protecting from side effects associated to chemo- and radiotherapy [97], though no biological evidence has been provided. In spite of these promising results, the paucity of well-controlled clinical trials, the poor bioavailability of curcumin and the limited effects reported by some investigators are currently a major limitation to the therapeutic use of curcumin.

### 4.3. Epigallocatechin Gallate

Epigallocatechin gallate (EGCG), the most active and major component of polyphenols in green tea, is known to be the principal contributor to the potential benefits of green tea to human health [98]. Hence, it is not surprising that EGCG and other tea catechins have been claimed of anticarcinogenic and antimutagenic activities [99]. The use of EGCG as a possible chemopreventive agent is supported by a number of studies regarding the ability of EGCG to modulate Nrf2-mediated cellular events [100, 101]. There is also substantial evidence that EGCG can display antisenescence effects, as observed in mesenchymal stem cells [101, 102].

#### 4.3.1. EGCG as Toxic Compound in Cancer

The cytoprotective effect of EGCG is apparently in contrast with a number of studies in cancer cells suggesting that induction of apoptosis could be the main mechanism of green tea to suppress cancer cell growth [103]. This paradoxical effect and the different effects shown in cancer versus primary cells could be related to their different metabolism including defects in the regulatory feedback that involves mTOR, p53, and AMPK [5] which are related to the epigenetic differences between cancer and normal cells [104, 105]. EGCG has been shown to be able to induce dose-dependent (5–80 *μ*M) apoptotic cell death in estrogen receptor- (ER-) independent breast cancer cells via an increased Bax to Bcl-2 protein ratio and p53 expression [106]. Moreover, EGCG appears to be critical for cancer cell metabolism due to the inhibition of mitochondrial functions and the generation of a starvation-like condition that activates AMPK and its downstream effects, including inhibition of mTOR signaling [107] and the activation of a sustained autophagic response that can promote autophagic cell death [108].

#### 4.3.2. EGCG as Senescence Inducer

Nontoxic concentrations (15 *μ*M) of EGCG shortened telomeres, increased SA-*β*-Gal staining, induced chromosomal abnormalities, and, most importantly, limited the lifespan of U937 monoblastoid leukemia and colon adenocarcinoma cell lines (HT29) [109]. Experiments in breast cancer (MCF-7) and promyelocytic leukemia (HL60) cell lines have confirmed an inhibitory activity on telomerase activity by EGCG [110]. Alterations in histone modifications, decreased methylation of hTERT promoter, and increased binding of the hTERT repressor E2F-1 at the promoter were proposed as mediators of the observed bioactivity [110].

#### 4.3.3. EGCG as Potential Senolytic

Although there are studies that have indicated EGCG as a senomorphic (suppressor of senescent phenotype) compound *in vitro* with potential lifespan-extending effects in animal models [111], there is currently no evidence that EGCG can exert senolytic activity in selected type of senescent cells.

#### 4.3.4. EGCG in Cancer Adjuvant Therapy

EGCG has also been proven to synergize with some anticancer agents and to ameliorate their deleterious side effects, which makes EGCG a suitable adjuvant in chemotherapy [112]. However, most of the studies on this topic are preclinical, and several limitations in terms of stability, efficacy, and bioavailability have currently hampered the application of EGCG in clinical settings [113]. There are contrasting results depending on the type of cancer and therapy. For example, EGCG was shown to provide regression of esophagitis in patients with unresectable stage III non-small-cell lung cancer under chemo- and radiotherapy [114]. Conversely, green tea polyphenols may have the potential to negate the therapeutic efficacy of the boronic acid-based synthetic anticancer drug bortezomib, thus suggesting that EGCG may be contraindicated during cancer therapy with bortezomib [115]. Finally, it is important to note that the concentrations of EGCG used *in vitro* (tens of micromolar) are usually far from levels observed in serum after drinking few cups of tea as biologically achievable concentrations were generally reported to be below 1 *μ*M [116].

### 4.4. Quercetin

Quercetin is a member of flavonoid found in many dietary plants such as apple, apricot, broccoli, Brussels sprout, cauliflower, grape, lettuce, onion, strawberry, tomato, and wolfberry [117]. Quercetin has been reported to have anti-inflammatory, antidiabetic, antiobesity, and anticancer activities [118, 119]. Quercetin is also widely known to exert antioxidative stress activity via activating Nrf2 signaling pathway [120–122]. It has been demonstrated that quercetin can display antisenescence activity in normal cells. Senescent fibroblasts treated with about 6 *μ*M of quercetin for 5 consecutive days were shown to restart proliferation compared to the control cultures [123].

#### 4.4.1. Quercetin as Toxic Compound in Cancer

There are several studies that propose the use of quercetin to induce apoptotic and nonapoptotic forms of cell death in cancer cells [124, 125]. Various mechanisms have been claimed to explain the ability of quercetin to bypass apoptotic resistance of cancer cells. Most of the studies report that quercetin can target antiapoptotic kinases and selective oncogenes (such as Mcl-1, Ras, MEK, and PI3K) or upregulate tumor suppressor genes (p53, p21), which lead to the selective elimination of cancer cells [126]. There is also evidence for an involvement of heat shock response proteins (HSP) in the toxicity of quercetin for cancer cells. Various quercetin-treated tumor cell lines were not induced to show aggregation of HSP70 in the nuclei in response to heat shock, resulting in apoptosis [127].

#### 4.4.2. Quercetin as Senescence Inducer

As shown for most Nrf2-activating compounds, the finding that, in certain circumstances, it is also possible to use quercetin to induce senescence in cancer cells is not surprising. Chronic administration of 25 *μ*M quercetin plus 10 *μ*M resveratrol was shown to induce a senescent-like growth arrest in human glioma cells [128]. The prosenescence activity of quercetin in the glioma cellular models is compatible, at least in part, with the inhibition of HDAC (histone deacetylases) [129]. Interestingly, this inhibitory activity on HDAC was not observed in normal astrocytes. Quercetin was also shown to activate and stabilize p53 by inhibiting its RNA degradation and protein ubiquitination in liver carcinoma cells (HepG2), thus promoting p21 expression and cyclin D1 suppression in favor of cell cycle arrest [130]. Hence, circumstances where p53 is not stabilized or where HDAC is over activated pave the way to a potential use of quercetin to induce senescence in cancer.

#### 4.4.3. Quercetin as Potential Senolytic

Quercetin (10 *μ*M) was proven to induce death in radiation-induced senescent endothelial cells and senescent bone marrow-derived mouse mesenchymal stem cells [38]. Conversely, quercetin was found to lack senolytic efficacy in senescent preadipocytes and mouse embryonic fibroblasts. The combination of quercetin with the anticancer drug dasatinib was shown to be effective as senolytic in several types of senescent cells [38]. In the context of cancer therapy, the potential of quercetin (or its combination with dasatinib or other compounds) to induce death in cancer cells after therapy-induced senescence should deserve appropriate investigation. This could be useful to reduce adverse effects of chemotherapy and cancer relapse, which are promoted by therapy-induced cellular senescence [42].

#### 4.4.4. Quercetin in Cancer Adjuvant Therapy

Excluding studies designed to test safety, availability, and metabolism of quercetin [131], its use in clinical trials as adjuvant therapy for cancer patients still need to be appropriately investigated. Interesting results have been observed regarding the modulation of cancer-related biomarkers in few patients with ovarian cancer and hepatoma [132]. Critical points that hamper the use of quercetin in these trials include the side effects of the pharmacological dose that need to be administered, the lack of specificity, and the identification of direct cellular targets.

### 4.5. Genistein

Genistein is an isoflavonoid compound present in some edible plants such as alfalfa, soybean, fava bean, psoralea, pea, green lentil, and lupine [133]. Genistein is known for antioxidant, anticancer, anti-inflammatory, antiobesity, and antidiabetes activities [134–136]. In addition, this compound can protect cells from injury, toxicity, and oxidative stress by activating Nrf2 [12, 137, 138]. At relatively low concentrations (1–10 *μ*M), genistein has been shown to delay senescence in vascular smooth muscle cells [139] and to enhance telomerase activity in prostate cancer cells [140].

#### 4.5.1. Genistein as Toxic Compound in Cancer

Genistein can induce apoptotic and nonapoptotic cell death in several models of cancer cells [141]. For example, in H460 non-small lung and MDA-MB-231 breast cancer cells as well as in HT29 colon cancer cells, genistein inhibits cell growth and induces apoptosis at concentration from 30 to 50 *μ*M [142–144]. Genistein at lower concentration (10 *μ*M) can also sensitize sarcoma and breast cancer cells to X-ray-induced cell death by inhibiting the double-strand break (DSB) repair pathways [145, 146].

#### 4.5.2. Genistein as Senescence Inducer

Numerous studies have shown that genistein can induce the expression of tumor suppressor genes p53, p21, and p16 in cancer [134, 147–150] that mediate cell cycle arrest and senescent response. It has also been reported that genistein at pharmacological concentrations (50 *μ*M) inhibited telomerase activity in brain [KNS60, U251MG(KO), and ONS76], ovarian (SKOV-3), breast (MCF-7), and prostate (DU-145, LNCaP) cancer cells [140, 151].

#### 4.5.3. Genistein as Potential Senolytic

There is no information about senolytic activity of genistein but its inhibitory effect on tyrosine kinase [152] (the same target of dasatinib) would deserve appropriate consideration.

#### 4.5.4. Genistein in Cancer Adjuvant Therapy

The contrasting results obtained in a relatively narrow range of concentration suggest that the use *in vivo* of this compound might deserve particular caution. Genistein aglycone can eventually stimulate tumor cell proliferation and growth in mice that exhibit a deficient immune system [153]. Moreover, epidemiological studies have shown an inverse correlation between genistein intake and breast cancer risk [153].

### 4.6. Resveratrol

Resveratrol is a naturally occurring polyphenolic compound present in grapes, mulberries, peanuts, and red wine. It has been identified as a cancer chemopreventive agent, based on its safety and efficacy in experimental models of carcinogenesis [154]. The antitumor activity of resveratrol has been attributed to the inhibition of diverse cellular events associated with tumor initiation, promotion, and progression [155]. Inhibition of carcinogenesis and the chemopreventive effects of resveratrol might be related to the induction of Nrf2-mediated protective pathways [156].

#### 4.6.1. Resveratrol as Toxic Compound in Cancer

*In vitro* studies suggest that resveratrol is able to induce growth inhibition and apoptosis in several tumor cell lines [157–159]. The IC_50_ value in five cell lines (Seg-1, HCE7, SW480, MCF7, and HL60) was attributed to be in the range of 70–150 *μ*M [160] and only three of these cell lines (MCF7, HL60, and Seg-1) started to show a significant reduction in cell viability at 50 *μ*M.

#### 4.6.2. Resveratrol as Senescence Inducer

Resveratrol represents also one of the most active natural compounds in inducing senescence in cancer cells, in particular at concentrations equal or below 50 *μ*M. The increase in the activity and expression of senescence-associated effectors (e.g., p53 and p21) was observed in various cancer cells treated with resveratrol. Resveratrol has shown to be able to exert SIRT1-dependent inhibitory effects on gastric cancer by inducing senescence in cellular models, as evidenced by the increased protein levels of inhibitors of CDKs (p21 and p16) and SA-*β*-Gal staining in resveratrol-treated samples [161]. The inhibitory effect on gastric cancer was also confirmed *in vivo* using a nude mice xenograft model. This effect was abrogated after SIRT1 (sirtuin 1) depletion probably through an indirect regulation of involved genes. Evidence of the involvement of senescence-associated effectors in the resveratrol-mediated antitumor action has been shown in many other tumor cell lines [162–164]. As it happens for some drugs, even resveratrol would seem to hijack the fate of tumor cells towards antiproliferative pathways depending on the dose of treatment and this phenomenon appears to be important also in cancer prevention [165]. In particular experimental settings, there is evidence that resveratrol may act as a potent senescence inducer. It has been shown that micromolar doses (10–50 *μ*M) of resveratrol-treatment in non-small-cell lung cancer cells can lead to a significant increase in SA-*β*-Gal staining and enhanced p53 and p21 expression, suggesting that the anticancer effect of resveratrol is largely attributable to the induction of senescence [166]. Similar concentrations of resveratrol have been effective in reducing the telomerase activity in MCF-7 breast cancer cells, probably affecting posttranscriptional phosphorylation and nuclear translocation of the catalytic subunit hTERT [167]. In accordance with these results, inhibition of transcriptional hTERT expression was proposed as a mechanism to explain resveratrol-mediated inhibition of human colorectal carcinoma cell proliferation [168]. All the above results highlight the ability of resveratrol to modulate different pathways related with the complex machinery of CS depending on the tumor types and treatment conditions.

#### 4.6.3. Resveratrol as Potential Senolytic

There is no reported senolytic activity for resveratrol. A recent high throughput screening of senotherapeutics in senescent Ercc1−/− mouse embryonic fibroblasts showed no effect (neither senolytic nor senomorphic) of resveratrol, but this could be due to the concentration tested (not clearly specified, but likely at 1 *μ*M as declared for other compounds) and the specific model used [41].

#### 4.6.4. Resveratrol in Cancer Adjuvant Therapy

Lack of specificity, efficacy, and poor bioavailability is the major limitation for the use of resveratrol as adjuvant therapy in cancer. While *in vitro* resveratrol seems to be highly effective in overcoming chemoresistance (at concentration of 25–50 *μ*M), for example, in the case of multiple myeloma cells [169], the clinical translation of these doses in clinical settings appears problematic. Indeed, an unacceptable safety profile and minimal efficacy were shown in a clinical trial performed with 5 g/day of SRT50 (a micronized oral formulation of resveratrol developed to improve bioavailability) combined with bortezomib in patients with relapsed/refractory multiple myeloma [170]. However, the same dose and formulation resulted safe when administered in patients with colorectal cancer and hepatic metastases [171], suggesting that a thorough patient cohort study should be defined before clinical applications.

### 4.7. Silybin

Silybin, a major active constituent of silymarin (extract of the milk thistle seeds), has been shown to have antioxidant and cytoprotective as well as antitumor effects. Moreover, several studies performed in *C. elgans* suggest that silybin may display antiaging activity, mainly based on counteracting age-related loss of proteostasis [172–174]. In analogy with other flavonoids, also in this case, the antioxidant and cytoprotective effects seem to be related to the activation of Nrf2 pathway [175].

#### 4.7.1. Silybin as Toxic Compound in Cancer

Silybin was found to induce growth inhibition and apoptosis in different human and murine tumor cell lines and to potentiate the effects of doxorubicin, cisplatin, and carboplatin *in vitro* [176–178]. Fewer studies have been conducted on the antitumor effect exerted by *in vivo* supplementation with silybin or silymarin. Most data on the *in vivo* effects of these compounds, confirming a general anticancer activity, have been drawn from studies done in mice treated with carcinogens or in nude mice bearing human xenografts [4].

#### 4.7.2. Silybin as Senescence Inducer

Study reported that IdB 1016 (silipide), a silybin-phosphatidylcholine complex with improved bioavailability, induced cellular senescence in mammary tumor cells of mice at 450 mg/Kg, as demonstrated by SA-*β*-gal staining in cancer tissues. According to the same study, this complex (at concentration in the range of 10–50 *μ*M) also induced cellular senescence and apoptosis in human breast SKBR3 cancer cell line, which were associated with increased expression of p53 [179].

#### 4.7.3. Silybin as Potential Senolytic

There is currently no evidence for a senolytic activity of silybin. However, the cooccurrence of markers of apoptosis and senescence in breast cancer cells treated with silybin [179] would suggest appropriate investigation in this field.

#### 4.7.4. Silybin in Cancer Adjuvant Therapy

Conversely to the lack of cancer tissue penetration observed in prostate cancer patients (receiving 13 g per day of silybin-phytosome) [180], administration of silybin-phosphatidylcholine, 2.8 g daily, 1 month before surgery, to patients with early breast cancer showed a selective accumulation of silybin in breast tumor tissue [181]. However, clear proof of clinical efficacy as adjuvant in cancer therapy is still lacking. A pilot study (administration of 2 g per day in 3 patients) in advanced hepatocellular carcinoma demonstrated the complete lack of benefits [182].

### 4.8. Phenethyl Isothiocyanate

Phenethyl isothiocyanate (PEITC) is a member of isothiocyanate distributed as gluconasturtiin in some cruciferous plants including broccoli, cabbage, cauliflower, horseradish, and watercress [105]. This compound has multiple pharmacological activities including anticancer activity [183]. It has been reported that PEITC exhibits antioxidant activity by affecting Nrf2 signaling pathway [11, 184].

#### 4.8.1. PEITC as Toxic Compound in Cancer

PEITC (at 5–10 *μ*M) induces apoptosis in several cell lines by a cancer cell-specific generation of ROS [185] that is related to mitochondrial deregulation and modulation of proteins like Bcl2, BID, BIM, and BAX, causing the release of cytochrome c into cytosol leading to apoptosis [183]. Other mechanisms by which PEITC induces apoptosis (at 50 *μ*M) include the increase of DDB2 (damaged DNA-binding protein 2) expression, as observed in colon cancer cells (HCT 116) *in vitro* and *in vivo* [186], as well as the activation of the extrinsic apoptotic pathway (death receptor-mediated apoptosis), as observed in oral and cervical cancer cells [187].

#### 4.8.2. PEITC as Senescence Inducer

Modulation of the senescence effectors p16, p53, and p21 as well as increased staining for SA-*β*-Gal by PEITC was observed in cancer cells at concentration from 4 *μ*M to 20 *μ*M [186, 188–190]. PEITC also downregulated telomerase in cervical cancer cells (HeLa) [189].

#### 4.8.3. PEITC as Potential Senolytic

The potential for PEITC as senolytic agent has been tested in radiation-induced senescent WI-38 fibroblasts without any evidence of selective death in normal versus senescent cells (LD50 ratio = 1) [191].

#### 4.8.4. Efficacy of PEITC in Cancer Adjuvant Therapy

Preclinical evidence suggests that combination of PEITC with conventional anticancer agents is also highly effective in improving overall efficacy [183]. There is a clinical trial showing that PEITC can be an inhibitor of lung carcinogenesis [192], but its relevance in adjuvant cancer therapy is still unknown.

### 4.9. Sulforaphane

Sulforaphane is one of the most potent phase II enzyme inducer isolated from edible cruciferous vegetables with potent activity against cancer progression [193]. This activity has been demonstrated at the level of chemoprevention, as well as at the level of therapy at various stages of cancer. Sulforaphane represents a strong activator of Nrf2-Keap1 signaling pathway, enabling Nrf2 to escape Keap1-dependent degradation and leading to stabilization and nuclear accumulation of Nrf2 [194]. Acting through the Nrf2 pathway, sulforaphane inhibited 7,12-dimethylbenz(a)anthracene-induced skin tumorigenesis in mice [195]. At the same time, the Nrf2 pathway seems to be involved in the sulforaphane-mediated protection from apoptosis in different cellular models [196, 197]. Oral administration of sulforaphane was able to inhibit DMBA-induced mammary carcinogenesis in rats [198]. In this animal model, an accumulation of sulforaphane metabolites, followed by an increased expression in NQO1 and HO-1 cytoprotective mRNAs, was observed in mammary gland after a single oral 150 *μ*M dose of sulforaphane. Interestingly, a local increase of SFN metabolites was observed in epithelial cells from human breast tissue after a single oral sulforaphane dose (200 *μ*M) in healthy women undergoing reduction mammoplasty. Hence, the specific intracellular accumulation and retention of this compound in mammary epithelium might contribute to protecting normal cells from tumor initiation and progression, though more large-scale clinical trials are needed to verify the effectiveness of sulforaphane as anticancer agent.

#### 4.9.1. Sulforaphane as Toxic Compound in Cancer

Sulforaphane has been defined as “hormetic” dietary compound, because of its ability to induce different/opposite biological effects at different doses [199]. Treatment of mesenchymal stem cells (MSC) with low doses of sulforaphane (0.25–1 *μ*M) increased cell proliferation protecting from apoptosis and senescence, and conversely higher doses (5–20 *μ*M) induced cytotoxicity together with HDAC inhibition and increasing number of apoptotic and senescent cells [199]. However, it has been frequently reported a different IC_50_ for normal and cancer cells. For example, IC_50_ values from 14.0 to 19.3 *μ*M were found in MCF-7, MDA-MB-231, and SK-BR-3 breast cancer cell lines, whereas the IC_50_ for normal human mammary epithelial cells was 81.24 *μ*M [200]. Sulforaphane-induced HDAC inhibition and induction of cell death has been shown in various cancer cells [201]. In colorectal cancer cells, sulforaphane treatment (15 *μ*M) induced alteration of histone acetylation status and a specific increase in acetylated histone H4 bound to the promoter region of *P21* leading at an increased p21Cip1/Waf1 protein expression [202]. Consistently, *in vivo* administration of sulforaphane inhibited HDAC activity in mouse colonic mucosa after six hours from the oral treatment with concomitant increase of acetylated H3 and H4 histone. Changes in histone acetylation status were also observed after long-term (10 weeks) administration of sulforaphane diet that resulted in augmented acetylated histones and p21 expression in the ileum, colon, prostate, and PBMC cells. Dietary sulforaphane was also able to suppress polyp formation in *Apc*^min^ mice [203]. In addition, changes in the histone modifications of the hTERT promoter and DNA demethylation of hTERT exon 1 were observed in human breast cancer cells in response to sulforaphane [204]. Acting as HDAC inhibitor, sulforaphane may be useful in the treatment of many types of cancer in which HDAC activity and hypoacetylation contribute to malignant progression.

#### 4.9.2. Sulforaphane as Senescence Inducer

As mentioned above, the dose and duration of sulforaphane treatment result in a divergent cell fate also in cancer cells. Sulforaphane at 5–10 *μ*M promotes cell cycle arrest, elevation in the levels of p21 and p27, and cellular senescence in breast cancer cells (MCF-7, MDA-MB-231, and SK-BR-3), whereas at the concentration of 20 *μ*M, apoptosis was induced [200]. The effects were mediated by upregulation of sixty microRNAs and downregulation of thirty-two microRNAs, global hypomethylation, and decreased levels of DNA methyltransferases (DNMT1, DNMT3B), as well as nitrooxidative stress, genotoxicity, and diminished AKT signaling. Transient sulforaphane exposure for up to 6 hours induced reversible G2/M growth arrest, while exposures of 12 to 72 hours resulted in irreversible G2/M arrest and apoptosis of human colon cancer cell line [205]. Cell cycle arrest in G1 phase and induction of key effector molecules related to cellular senescence, such as p21, p27, Rb, and PAI-1, has been observed by treatment with sulforaphane of adipocytes at early stage of differentiation [206].

#### 4.9.3. Sulforaphane as Potential Senolytic

There is currently no evidence of senolytic effects of sulforaphane.

#### 4.9.4. Efficacy of Sulforaphane in Cancer Adjuvant Therapy

Sulforaphane is considered a good candidate in adjuvant therapy of cancer due to its proapoptotic, antiangiogenesis, and antimetastasis activities shown in preclinical settings [207]. However, clinical studies in men with recurrent prostate cancer have shown limited efficacy with significant effects only on secondary endpoints [208, 209].

### 4.10. Triptolide

Triptolide is a natural diterpenoid abundant in thunder god vine (*Tripterygium wilfordii*). It has gained importance because of its potential for prevention and treatment of cancer [210]. Triptolide is able to induce toxic cellular effects, which induce Nrf2 and its target genes, as it has been shown in hepatic cell lines [104].

#### 4.10.1. Triptolide as Toxic Compound in Cancer

Triptolide (50–100 nM) is able to decrease mitochondrial respiration and increase ROS and apoptosis in p53-deficient non-small-cell lung cancer and consequently to upregulate Nrf2 and its target gene HO-1 and NQO1 [10]. This compound was shown to induce apoptosis and cell cycle arrest in various cancers by targeting the p53/p21 and BCL-2 pathway [211–213].

Conversely, in resistant myeloid leukemia cell lines, triptolide enhanced the sensitivity to doxorubicin-induced and imatinib-induced apoptosis through a downregulation of Nrf2 and its target genes [214].

#### 4.10.2. Triptolide as Senescence Inducer

Triptolide has the ability to induce senescence. Treatment of liver cancer cells (HepG2) with nanomolar concentrations of triptolide (2.5–10 nM) induced senescence via Akt and hTERT pathway [215]. Triptolide (3 nM) induced senescence of primary prostate adenocarcinoma cells, as demonstrated by SA-*β*-gal activity [216].

#### 4.10.3. Triptolide as Potential Senolytic

There is currently no evidence of senolytic effects of triptolide. Triptolide (0.25 mg/kg i.v., twice weekly for 1, 2, and 3 months) is able to mitigate radiation-induced pulmonary fibrosis in rats [217] but conversely to senolytic drug, which can be given when fibrosis is permanent [218]; triptolide beneficial effects have been demonstrated when given before irradiation. Anyway, further studies could be performed in this area due to the important proapoptotic effects shown by triptolide in cancer cells.

#### 4.10.4. Triptolide in Cancer Adjuvant Therapy

A phase I and pharmacological study of F60008 (a semisynthetic derivate of triptolide, which is converted to triptolide) given intravenously in patients with advanced solid tumors displayed various adverse effects without any clear proof of efficacy [219].

### 4.11. Allicin

Allicin, an organosulfur compound, is mainly present in garlic (*Allium sativum*). The compound is reported to have antimicrobial, anticancer, and cardioprotective activities [220]. Allicin inhibited lipopolysaccharide-induced vascular oxidative stress and inflammation in human umbilical vein endothelial cells, which were associated with activation of Nrf2 and reduction of TNF-*α* (tumor necrosis factor *α*) and IL-8 (interleukin 8) production [221]. By activating Nrf2, allicin protected spinal cord tissue from traumatic injury in rats [222].

#### 4.11.1. Allicin as Toxic Compound in Cancer

Allicin (10–30 *μ*M) reduced cell viability and proliferation in several mammalian lines with higher efficacy to induce apoptosis in 3T3 and MCF-7 cell lines [223]. Treatment of liver cancer cells with allicin induced apoptotic cell death via p53 modulation [224].

#### 4.11.2. Allicin as Senescence Inducer

Allicin has been shown to inhibit telomerase activity and to induce apoptosis in gastric cancer adenocarcinoma cells (SGC-7901) [225]. While conventional senescent markers have not been measured in this study, cells treated with allicin (100 *μ*M) showed typical morphological changes (enlarged and irregular) that have been reported in several models of senescence. However, cells treated with allicin underwent rapid apoptosis after morphological changes, and these changes were more likely related to cell death events rather than senescence.

#### 4.11.3. Allicin as Potential Senolytic

Allicin has not been investigated in this area.

#### 4.11.4. Allicin in Cancer Adjuvant Therapy

Clinical trial with allicin as adjuvant in cancer therapy is still lacking. There is a report of partial efficacy (mild increase of apoptosis in cancer tissues) of a local application of allicin, via gastroscopy (48 h before surgical intervention), in patients with progressive gastric carcinoma.

### 4.12. Berberine

Berberine is a naturally occurring isoquinoline alkaloid present in barberry (*Berberis vulgaris*), tree turmeric (*B. aristata*), oregon grape (*B. aquifolium*), goldenseal (*Hydrastis canadensis*), and goldethread (*Coptis chinensis*) [226]. Berberine has been shown to possess a wide range of pharmacological activities [227], including antidiabetic, antihyperlipidemic, antiarrhythmic, and antioxidat activities that find a common rationale in the upregulation of Nrf2-related pathways [228, 229].

#### 4.12.1. Berberine as Toxic Compound in Cancer

Berberine displays hormetic effects “in vitro.” It has been shown that berberine at low-dose range (1.25 ~ 5 *μ*M) can promote cell proliferation while at high-dose range (10 ~ 80 *μ*M) can inhibit cell proliferation [230]. *In vitro* treatment with berberine can inhibit cell growth and induce cell cycle arrest and apoptosis (IC_50_ from 7 to 20 *μ*M) of various cancer cells, for example, prostatic, gastrointestinal, hepatic, and mammary human cancer cells (reviewed in [231]), as well as skin- [232] and hematological-derived cancer cells [233]. Activation of AMPK, inhibition of mTOR pathway, and induction of apoptosis or autophagic cell death are the best-characterized cascade of events by which berberine exerts anticancer activity [234, 235].

#### 4.12.2. Berberine as Senescence Inducer

However, there is a series of scientific evidence about the ability of berberine to exert cell type-specific effects that, in certain circumstances, include cell cycle arrest and induction of a senescent-like phenotype. Indeed, chronic treatment with berberine (15 *μ*M) for one week was shown to induce senescence in human glioblastoma cells by downregulation of EGFR-MEK-ERK signaling pathway [236]. Moreover, the antitumor effects of berberine and berberine derivatives in human HER-2/neu overexpressing breast cancer cells are mediated not only by apoptotic cell death but also by increased expression of p53, p21, p16, and PAI-1 mRNAs, thus suggesting that the mechanism of action of berberine may also include the induction of CS [237]. Another potential mechanism that could explain this role of berberine in CS regards the inhibition of telomerase activity forming a G-quadruplex with telomeric DNA [238]. Finally, treatment of promyelocytic leukemia HL-60 cell line with 150 *μ*M berberine induced a time-dependent reduction in the activity of telomerase [239].

Berberine was also shown to trigger the transcriptional activity and the inhibition of the degradation of p53 in human breast cancer MCF7 cells [240]. All these observations suggest that berberine is another example of natural Nrf2-activating compound that exerts different and even contrasting, that is, gerosupressive [241] and prosenescence [236, 237], effects likely depending on cell type, time of exposure, and dosage. It is important to consider that most, if not all, studies *in vitro* with berberine tested doses in the micromolar range which is far higher than levels achievable in blood plasma after oral dosing. These observations prompt further investigation to clarify the conditions that might allow to use safely berberine in prosenescence therapy for cancer.

#### 4.12.3. Berberine as Potential Senolytic

A compound with the ability to modulate FLIP in senescent cells may potentially be used as a senolytic drug. Berberine is among those compounds that modulates FLIP and has been included in a recent patent as potential senolytic [242]. Studies are currently in progress around this topic.

#### 4.12.4. Berberine in Cancer Adjuvant Therapy

Recent applications related to berberine's possible therapeutic use are focused on metabolic syndrome, type 2 diabetes, and dyslipidemia. However, the use of berberine as adjuvant therapy in cancer appears to be promising as well. Berberine reduced radiation-induced lung injury (RILI) and pulmonary fibrosis in non-small-cell lung cancer (NSCLC) patients treated with radiotherapy [243]. Moreover, there is evidence that oral administration of berberine can reduce the familial adenomatous polyposis patients' polyp size [244]. Additional trial with berberine as chemopreventive agent as well as in reducing recurrence rates of colorectal adenoma (CRA) is currently ongoing.

### 4.13. Piperlongumine

Piperlongumine is a natural alkaloid isolated from the long pepper. It is a potent inducer of Nrf2 response and of its target genes including heme oxygenase-1 (HO-1) [50]. Interestingly, HO-1 has antitumor functions in cancer cells, but cytoprotective functions in normal cells.

#### 4.13.1. Piperlongumine as Toxic Compound in Cancer

Piperlongumine displays a high degree of selective toxicity to cancer cells. It has been identified as strong inhibitor (IC_50_ = 1.7 *μ*M) of signal transducer and activator of transcription 3 (STAT3) by a recent high throughput drug-repository screening [245]. STAT3 is a validated drug target for cancer therapy and thus it is not surprising that piperlongumine was found to be able to induce apoptosis at low doses (IC_50_ from 0.16 up to 5.1 *μ*M) in multiple breast cancer cell lines having increased STAT3. This proapoptotic activity is associated with the modulation of several antiapoptotic genes including Bcl-2, BcL-xL, survivin, X-linked inhibitor of apoptosis (XIAP), and cellular inhibitor of apoptosis proteins (cIAP). Alone and in combination with cisplatin, piperlongumine (2.5–15 *μ*M) is able to dysregulate the oxidative stress response and kill head and neck cancer cells independently by their p53 mutational status [246] as well as a multitude of pancreatic, kidney, breast, lung, and pancreatic cell lines (Panc1, L3.6pL, A549, kidney, and SKBR3) [247]. In human oral squamous cell carcinoma, piperlongumine induces increased ROS and subsequent caspase-dependent apoptosis at 7.5–10 *μ*M [248]. However, another study found no evidence of dose-response relationship between cellular ROS, induced by piperlongumine, and its cytotoxicity [249], thus suggesting the presence of different mechanisms related to induction of cell death.

#### 4.13.2. Piperlongumine as Senescence Inducer

Piperlongumine has been shown to suppress proliferation and to induce p21-mediated senescence (2.5–7.5 *μ*M) [248] in human oral squamous cell carcinoma cells.

#### 4.13.3. Piperlongumine as Potential Senolytic

A recent library screening for compounds with senolytic activity identified piperlongumine as a promising compound. It has been shown to preferentially induce cell death in irradiation, replicative, and oncogene-induced senescent WI-38 fibroblasts (EC_50_ 6–8 *μ*M) compared to nonsenescent fibroblasts (EC_50_ 20 *μ*M) [46]. However, apoptotic mechanisms of piperlongumine in senescent cells were found to be independent by the generation of ROS [46].

#### 4.13.4. Piperlongumine in Cancer Adjuvant Therapy

Piperlongumine was found to be nontoxic in mice up to a dose of 30 mg/kg/day for 14 days and caused regression of breast cancer cell line xenografts in nude mice. These results, in addition to the recently discovered activity as senolytic compound, hold promises for a potential translation in human trials.

### 4.14. Fisetin

Fisetin is an organic flavonoid present in numerous fruits and vegetables such as strawberries, mangoes, and cucumbers that exhibits antioxidant, neurotrophic, anti-inflammatory, and anticancer effects. Attention on fisetin in the context of aging research and chemopreventive therapy is mostly related to its ability to increase transcriptional activity of Nrf2 [250] and its target gene HO-1 [251] and also to inhibit the activity of mTOR kinase [252].

#### 4.14.1. Fisetin as Toxic Compound in Cancer

In prostate cancer cells with upregulated activity of pathway upstream mTOR, high concentration of fisetin (40 *μ*M and above) induces autophagic cell death [253]. Death induction in monocytic leukemia cells by fisetin (IC_50_ = 50 *μ*M) was mediated by an increase in NO resulting in the inhibition of the downstream pathways of mTOR, double-strand DNA breaks, and caspase activation [254]. Fisetin can induce apoptosis and suppress the growth of colon cancer cells (HCT116 and HT29) with an IC_50_ comprised from 50 to 132 *μ*M after 72 h of exposure [255], and similar effects were observed in prostate cancer cells (PrEC, LNCaP, and CWR22Rv1) with an IC_50_ comprised from 20 to 60 *μ*M after 48 h of exposure.

#### 4.14.2. Fisetin as Senescence Inducer

The mechanism of accelerated cellular senescence was not observed among those involved in the antiproliferative effects of fisetin (1–50 *μ*M) in PC3 or lymph node carcinoma of the prostate (LNCaP) cells [256]. There is no further investigation about a potential prosenescence effect of fisetin.

#### 4.14.3. Fisetin as Potential Senolytic

Fisetin selectively induces apoptosis (at 5–10 *μ*M) in senescent, but not in proliferating, HUVECs. However, it is not senolytic in senescent IMR90 fibroblasts or in primary human preadipocytes [44].

#### 4.14.4. Fisetin in Cancer Adjuvant Therapy

Although preclinical data appear to be convincing, well-designed clinical trials in humans are needed to conclusively determine the efficacy across various cancers as well as senolytic adjuvant therapy.

### 4.15. Phloretin

Phloretin is a dihydrochalcone flavonoid, which can be found in apple tree leaves. Phloretin has been shown to protect hepatocytes against oxidative stress [58] as well as HEI-OC1 auditory cells against cisplatin-induced apoptosis [51] by upregulating Nrf2 defensive pathway. Importantly, the cytoprotective effects of phloretin were also observed at relatively low doses (2.5–5 *μ*M) also in H9c2 cardiomyoblasts exposed to arsenic trioxide, a drug used in the treatment of acute promyelocytic leukemia that is associated to cardiotoxic side effects [257].

#### 4.15.1. Phloretin as Toxic Compound in Cancer

Phloretin is known to inhibit glucose transporter (GLUT) 2, a process which results in the induction of apoptosis in cells with high metabolic requirement, as shown in human liver cancer cells HepG2 treated with 200 *μ*M phloretin [258]. At the dose of 10 mg/kg, phloretin was found to exert antitumor effects in immune deficiency mice carrying a HepG2 xenograft [258].

Moreover, phloretin was shown to induce apoptosis of non-small-cell lung cancer (NSCLC) cell line A549, Calu-1, H838, and H520 (IC_50_ approx. from 50 to 100 *μ*M) through deregulation of Bcl-2 [259] and other ROS-related pathways, such as P38 MAPK and JNK1/2 [260] which are related to the rise of ROS. Interestingly, the anticancer effects were enhanced in presence of cisplatin, which suggest a potential in adjuvant cancer therapy. Similar proapoptotic effects, associated with increased ROS and ROS-related pathways, were observed after treatment with very high (200–300 *μ*M) concentrations of phloretin [261].

#### 4.15.2. Phloretin as Senescence Inducer

While there is evidence that phloretin can induce cell cycle arrest in cancer cells [261], this process seems to be unrelated to senescence induction as there are no clear data about the possibility to induce senescence with phloretin.

#### 4.15.3. Phloretin as Potential Senolytic

Phloretin at 50 *μ*M was found to specifically reduce the viability of therapy-induced senescent lymphoma cells [45]. These cells were also shown to be sensitive to another blocker of glucose transporters, cytochalasin B, thus suggesting that the mechanism by which phloretin induces cell death in senescent cells is related to their increased metabolic requirement.

#### 4.15.4. Phloretin in Cancer Adjuvant Therapy

The recent observation related to the senolytic activity of phloretin as well as its potential in combination with withaferin A to suppress gefitinib-resistant adenocarcinoma cell line growth [262] appears to be promising therapeutic strategy to overcome the occurrence of cancer relapse and the resistance to chemotherapy. However, these senescent and glucose-targeting therapeutic strategies have still not been tested in clinical settings.

## 5. Mechanisms Mediating the Cytoprotective, Cytotoxic, or Prosenescence Effects of Nrf2-Activating Compounds

Activation of Nrf2-regulated cytoprotective response could provide a selective advantage to tumor cells which is clearly in contrast to the majority of effects described in the above chapters. In line with the hypothesis that Nrf2 response is an advantage for cancer cells, some types of polyphenols (usually belonging to the flavone class) have been shown to sensitize different cancer cells to chemotherapy via an inhibitory activity on Nrf2 signaling pathway. These compounds include luteolin [263], chrysin [264], and apigenin [265] which, in turn, was shown to induce senescence in IMR-90 cells [266]. These results might form a good rationale to use these compounds as adjuvant in cancer therapy. However, a multitude of data, exposed in the chapters above, supports the opposite concept that most polyphenols and other natural bioactive compounds activating the Nrf2 pathway can display cytotoxic effects or promote senescence in cancer cells. This dichotomy could be explained by the activity of pathways unrelated to Nrf2. Moreover, tissue and cell type specificity of Nrf2 downstream targets appear to be still poorly understood and the range of stress-response phenotypes observed when components of the pathway are genetically disrupted in mice is not completely explained. Keap1 KO mice hepatocytes experience a different signaling and gene expression compared with controls treated with an Nrf2-inducing agent [267]. Some of the induced factors can even antagonize Nrf2 thus suggesting that in the presence of unrepaired damage, such as in cancer cells, the complex response to Nrf2 includes damage-sensing factors that may activate apoptotic or senescence mechanisms. Cytoprotective effects of Nrf2 against oxidative stress are related to the presence of a functional aryl hydrocarbon receptor (AhR), a transcription factor that display pleiotropic activity in the context of carcinogenesis [13] and while some AhR ligands can suppress senescence acting as tumor promoters [268], recent work suggests that Ahr gene can function as a tumor suppressor gene by inhibiting cell proliferation and promoting senescent-like phenotype thus counteracting cancer progression [269–271]. Bidirectional interactions of Nrf2 and AhR have been reported, thus suggesting that Nrf2 can directly modulate AhR signaling [272]. This signaling network includes Cyp1A1 and Cyp1B1 that have been reported to induce upregulation of the cyclin-dependent kinase inhibitors p27 and p21 [273] that are regarded as key effectors of cellular senescence.

Another interaction of Nrf2 that deserves attention in the context of cellular senescence regards p53. The transcription of some Nrf2 target genes involved in the antioxidant response can be suppressed by p53 [274], but their mutual interaction still remains unclear. It has been reported the Nrf2 can increase the expression of the p53 inhibitor mouse double minute 2 homolog (MDM2), which is an ARE-regulated target gene [275]. However, p53 can be stabilized by Nrf2 target genes, that is, NQO1, suggesting both a positive and negative coregulation between p53 and Nrf2 [274] that is likely affected by the specific genomic and epigenomic profile of the target cell as well as by the duration of the stress. Epigenetic changes induced by natural compounds targeting Nrf2 could be likely involved in this process. For example, EGCG, a known Nrf2-activating polyphenol, can reduce the expression of miRNAs that target and suppress p53 [276], while epigenetic depression of one of these miRNAs (miR-200a) was reported to contribute to the dysregulation of Nrf2 activity in breast cancer [277].

Nrf2 can also interact downstream of p53 with its target gene p21 [64] or with p16 pathway by activating Notch-1 signaling. One or more functional ARE sequences exist in the promoter of Notch1, thus it is not surprising that Notch1 signaling can be triggered by Nrf2 [14]. Interestingly, Notch-1 may act as an oncogene or a tumor suppressor gene even within the same tumor type, and recently, it has been implicated in induction of cellular senescence mediated by p16 [278] and p21 [279].

Finally, the Jun dimerization protein 2 (JDP2), an important player in the senescence program [280], has a critical role as a cofactor for Nrf2 in the regulation of the antioxidant-responsive genes and production of ROS [281].

**Figure 2:**
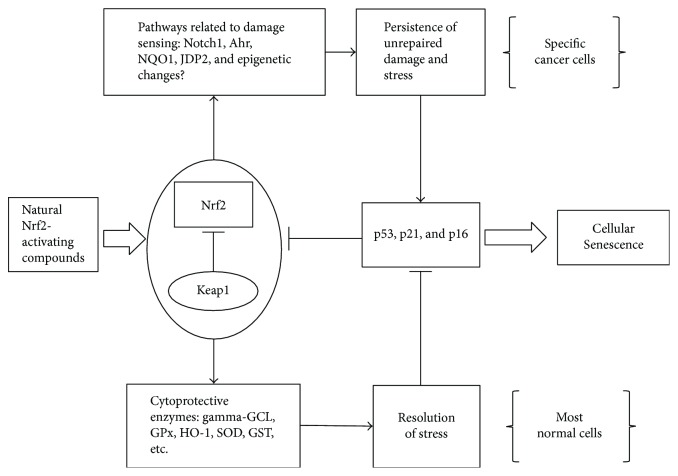
Potential mechanisms leading to senescence by NRF2-activating compounds in cancer cells. The response under NRF2 signaling involve the activation of glutamylcysteine ligase (*γ*-GCL), glutathione peroxidase (GPx), heme oxygenase 1 (HO-1), superoxide dismutase (SOD), glutathione S-transferase (GST), and many other enzymes involved in the antioxidant cytoprotective response that lead to suppression of senescence-related pathways (i.e., p53, p21, and p16). However, this response include and interact with additional genes, such as Notch-1, NADPH-quinone oxidoreductase (NQO1), the aryl hydrocarbon receptor (AhR), the Jun dimerization protein 2 (JDP2), and perhaps epigenetic changes that may be involved in sensing stress and damage and that are known to participate in processes leading to cellular senescence. In the case of (particular) cancer cells, the persistence of unresolved damage can eventually lead these pathways to the reactivation of the senescence program.

These data point towards a possible extension of Nrf2 to a more complex response that include damage-sensing prosenescence pathways, likely activated with a different timing in the case of persistent damage (Figure 2). However, it is also important to consider the multitarget ability of natural compounds, which might be able to interfere with senescence or apoptosis-related pathways, usually not directly related to Nrf2. These include multiple targets that have been frequently observed to be altered in cancer and senescent cells and that form the rationale to explain the different response from normal cells to the studied compounds [5]. Among the most critical targets that are noteworthy to mention are as follows:

- The reprogrammed metabolic pathway in cancer (Warburg effect or aerobic glycolysis) and some therapy-induced senescent cells, consisting in the switch of normal metabolism to support proliferation of cancer cells or production of SASP in senescent cells targeting glycolytic and other metabolic pathways, makes some cancer [282] and senescent cells [45] more susceptible to cell death than normal cells
- The defects in checkpoint kinases and repair genes that make cancer cells more susceptible to cell death following HDAC inhibition [104]
- The upregulation of antiapoptotic factors including Bcl-2/Bcl-xL and others mentioned above (shared by some cancer and senescent cells) which makes these cells susceptible to selective inhibitor
- The inhibition of autophagy (as consequence of high PI3K/Akt/mTor signaling) that makes cancer cells susceptible to cell death by AMPK inhibitors and starvation [283]
- The upregulation of both ROS and stress response pathway in cancer cells which make them susceptible to cytostasis by antioxidant treatment (due to unbalanced stress response) [284]

## 6. Nrf2-Activating Compounds and the Clearance of Senescent Cells

Senescent cells are an important source of inflammatory factors (SASP-related factors) for tumor progression [19] and can promote the development of cancer in the surrounding environment. In certain cases, it has been hypothesized that senescent cancer cells might even be able to overcome the “permanent” cell cycle arrest, revert their phenotype, and restart proliferation, thus suggesting that senescence may be used as therapy escape system by some cancer cells [16]. Taking into account that senescent cells can display long-term survival and resistance to apoptosis [285], finding ways to eliminate senescent cells might deserve a great impact on future adjuvant therapeutic strategies against cancer. This kind of senolytic adjuvant therapy could be important to reduce the incidence of relapses and the adverse effects of the therapy, as recently demonstrated in animal models [42]. In the last five years, at least 12 compounds have been identified as agents with senolytic activity *in vitro*, and more than half of them have been shown to be effective *in vivo* in animal models. Four of these candidate senolytics are natural bioactive compounds that have been described in the chapters above, namely, fisetin, quercetin, piperlongumine, and phloretin, and the estimation of their IC_50_ for senescent and nonsenescent cells is described in Table 1. The finding that some natural compounds that can be found in fruit and vegetables have been found to reduce specifically the viability of senescent cells is remarkable. Apparently, this could improve and accelerate the translation of these findings into clinical trials, as these compounds are available as supplement or included in extracts that are commercially available. However, there are some limitations that deserve to be discussed. First of all, it is important to bear in mind that a natural compound is not necessarily less toxic than a synthetic compound, especially when the doses required to achieve a therapeutic efficacy are high. If we look at the estimated IC_50_ reported in Table 1, it appears that the differences between the IC_50_ of nonsenescent and senescent cells are comprised in a relatively narrow range and that their ratio is comprised from 2 to 3. This means that at high dosage, these compounds could be toxic also for normal cells especially assuming a nonuniform distribution through the body. Moreover, experiments *in vitro* cannot take into account the effects of multitude of metabolites that originates from a bioactive compound as a consequence of the microflora and the enzymatic activity of our body. In the case these compounds should be used as senolytics, the therapeutic regimen would consist of a single or few more treatments with very high doses. In the case of quercetin, there is no example of senolytic therapy given alone, but its combination with dasatinib has been tested in mice. The combination of dasatinib 5 mg/Kg and quercetin 50 mg/Kg has been given as weekly single dose (by gavage) in ERRC1^−/Δ^ mice for several weeks [38] as well as in the form of single monthly dose (dasatinib 5 mg/Kg and quercetin 10 mg/Kg) for 3 months in normal mice [39] with several reported benefits and without any report of side effects. A similar dose in humans would approximately correspond to a dose around 1.5–2 g, while common oral dosage of quercetin supplements is in the range 50–500 mg. Moreover, when 2 g/day (corresponding to 25 and 36 mg/Kg for individuals of 80 and 55 Kg body weight, resp.) was given to healthy men, the serum quercetin concentration was found to be 5 *μ*M [286], which is below the IC_50_ observed for senescent cells *in vitro*Table 1. In addition, the efficacy of quercetin as senolytic has been proven only in irradiation-induced senescent HUVEC while there is no evidence of this activity for any other model of senescence.

Also, fisetin displays a restricted activity that is limited to irradiation-induced senescent HUVEC at concentration higher than those reported for quercetin. If the IC_50_ of fisetin in vitro is 3-fold, those described for quercetin, we should expect senolytic doses that are very near to a toxic dosage. In mice, the LD_50_ dose was 180 mg/Kg given intravenously, which may suggest that doses above 500 mg in humans may be unsafe. For piperlongumine and phloretin, there is not a clear LD_50_. However, piperlongumine displays senolytic activity at concentration below 10 *μ*M in vitro which may suggest a potential to achieve these concentrations *in vivo*.

Anyway, at this moment, it is unclear if bioactive natural compounds can be safely used as adjuvant senolytic agents after cancer therapy. Moreover, it is unclear why it should be preferred a natural compound to a peptide (FOXO4-DRI) or a drug (Navitoclax) that have a substantially higher ratio between the respective IC_50_ of nonsenescent to senescent cells [43, 287] and thus are more likely, at least theoretically, to display less side effects at senolytic doses.

## 7. Concluding Remarks

CS likely evolved as a tumor-suppressing mechanism to prevent proliferation of cells that are at risk for acquiring potentially hazardous and transforming mutations. The possibility that putative cytoprotective natural compounds can reactivate pathways that induce apoptosis or senescence in cancer cells or that display senolytic activity appears a realistic perspective *in vitro* and a promising field of research. Emerging evidence has demonstrated that therapy-induced senescence can be achieved at much lower doses of chemotherapy and that it is a critical mechanism through which many anticancer agents inhibit the growth of tumor cells [288]. However, there are still lots of problems that need to be addressed in planning clinical trials. The first question that needs to be addressed is can we efficiently use adjuvant natural compounds to reduce the dose of chemotherapy while keeping the same efficacy and/or reducing undesired side effects associated with the therapy. The most likely answer is that it may depend on the cancer type and on the therapy scheme. In clear antagonism with excellent in vitro findings, most clinical trials performed up to now show extremely limited efficacy of any adjuvant therapy with these bioactive compounds (Table 2**)**. There is already some (but limited) evidence that adjustments in the therapy scheme, especially in the case of curcumin, might help to achieve promising results. However, most applications are directed to elucidate the safety and bioavailability of the compound in oncologic patients. Even though a close correlation with the induction of CS has not been directly highlighted, some biological effects have been demonstrated as a direct effect of curcumin treatment.

In spite of these promises, we should acknowledge that now the benefit of the use of nutraceuticals as adjuvant in cancer therapies seems to be limited by a number of factors. These include issues related to the effective bioavailability of such active compound (in safety condition of treatment) but also concerning the heterogeneity of the population cohort in terms of age, type of cancer, comorbidity, and schedule of treatment. There are always concerns that the concentrations frequently used *in vitro* are excessive (tens to thousands of micromolar) and may not therefore reflect what happens with more physiological exposures (nanomolar ranges). For example, even if most of published studies in cell culture systems used 10–100 *μ*M of EGCG [5], the blood level of EGCG after consumption of 2-3 cups of green tea was reported to be 0.1–0.6 *μ*M [116]. Similarly, for berberine, in spite of the use of micromolar ranges in cell culture, the C max in humans after an oral dose of 400 mg was reported to be 0.1 nM [289], while higher doses (above 0.9 g/day) were shown to induce gastrointestinal side effects (Table 2). Moreover, the oxygen partial pressure in a cell culture system is much higher than that in the blood or tissues and the metabolism that occurs after (a limited) intestinal absorption suggests that the real “actors” are likely to be metabolites rather than the original compound tested in cell culture. Last, but not least, the possibility that these Nrf2-activating compounds can promote an antioxidant cytoprotective response in cancer cells thus aggravating resistance to chemotherapy should not be under evaluated. This problem could be afforded with more studies around the complex response of Nrf2 pathways in presence of cancer-specific alterations so that a personalized strategy can be developed. Moreover, induction of CS does not ensure that these cells can be cleared off by the immune system and they might eventually use senescence as a system to escape death with the potential to revert their phenotype later in time. This problem might be potentially avoided with the recent development of senolytics, which include quercetin, fisetin, piperlongumine, and phloretin among natural bioactive compounds. These senolytics may be useful in the case cancer cells may use senescence as an escape strategy from therapy as well as to reduce side effects of therapy. Studies on senolytic compounds open the feasibility of a new therapeutic scheme with a single dose of the compound after radio- or chemotherapy without the needs for escalation doses and continuous treatments (Figure 1). Overall, combining therapies with natural compounds with the aims not only to induce senescence in cancer cells but also to clear off from the organism senescent cells appear as a promising strategy in this field.

## Figures and Tables

**Table 1:** Estimated IC_50_ for senescent and nonsenescent cells of natural bioactive compounds with reported senolytic activity observed in vitro after the exposure time reported in brackets.

IC_50_	Senescent cells							Nonsenescent cells					Reference
Compound	IRS IMR-90	IRS WI-38	RIS WI-38	OIS WI-38	IRS HUVEC	IRS ADP	TIS LYMP	IMR-90	WI-38	HUVEC	ADP	LYMP	
Fisetin	50 *μ*M (72 h)				**30 *μ*M (72 h)**	>>60 *μ*M^∗^ (72 h)		>50 *μ*M (72 h)		>60 *μ*M^∗^ (72 h)	>>60 *μ*M^∗^ (72 h)		[44]
Quercetin					**10 *μ*M (72 h)**	>50 *μ*M (72 h)				30 *μ*M (72 h)	>50 *μ*M (72 h)		[38]
Piperlongumine		**7.97 *μ*M (72 h)**	**6.24 *μ*M (72 h)**	**7.09 *μ*M (72 h)**					20.3 *μ*M (72 h)				[46]
Phloretin							**50 *μ*M** ^**#**^ **(120** h**)**					>>50 *μ*M^#^ (120 h)	[45]

^∗^The highest concentration tested in the respective reference (IC_50_ not measured); IRS = irradiation-induced senescence; OIS = oncogene-induced senescence; RIS = replicative-induced senescence; ADP = preadipocytes; LYMP = lymphoma; ^#^unique dose studied. The IC_50_ highlighted in bold are those supporting a senolytic activity (based on the difference with nonsenescent cells).

**Table 2:** Clinical trials of adjuvant (or alternative) therapies with natural bioactive compounds in cancer.

*n*	Compound	Dose	Drug^∗^	Patients and scheme	Results of the trial	Ref.	Established link with the drug and induction of cellular senescence^∗∗^
1	Tocotrienols	200 mg twice per day of tocotrienol-rich fraction	Tamoxifen	Double-blinded placebo-controlled trial in 240 women with early breast cancer subdivided in 2 groups: tocotrienol-rich fraction (TRF) plus tamoxifen and placebo plus tamoxifen	No association between adjuvant tocotrienol therapy and breast cancer-specific survival (risk of mortality due to breast cancer was 60% lower in tocotrienol group but not statistically significant)	[81]	[290]

2	Curcumin	Tablets containing 500 mg Meriva, a proprietary lecithin delivery system containing 100 mg curcuminoids (33 parts of curcumin, 8 parts of demethoxycurcumin and 1 part of bis-demethoxycurcumin)	Patients were under different chemotherapy or radiotherapy regimen	A controlled study on the possibility to alleviate adverse effects of cancer treatment with Meriva. Half of patients (*n* = 80) received Meriva plus the “best available treatment” and the other half (*n* = 80, control groups) received only the “best available treatment”	Results showed that lecithinized curcumin might alleviate the burden of side effects associated to chemo- and radiotherapy	[97]	—

2	Curcumin	2.0 grams of curcumin or placebo orally three times per day (i.e., 6.0 grams daily)	Radiotherapy	Randomized, double-blind, placebo-controlled clinical trial to assess the ability of curcumin to reduce radiation dermatitis severity in 30 breast cancer patients	Curcumin significantly reduced the severity of radiation dermatitis (fewer moist desquamation)	[291]	[292]

2	Curcumin	Oral administration of highly bioavailable curcumin (Theracurmin) containing 200 mg or 400 mg of curcumin	Gemcitabine-based chemotherapy	A phase I clinical study on 16 pancreatic or biliary tract cancer patients who failed standard chemotherapy	No unexpected adverse events were observed and 3 patients safely continued Theracurmin administration for >9 months	[293]	[294]

2	Curcumin	360 mg curcumin three times daily presurgery (10–30 days)	Radiotherapy, chemotherapy, chemoradiotherapy, or no additional therapy	A placebo-controlled clinical trial randomized 126 patients with colorectal cancer to either receive curcumin or placebo	Curcumin administration increased body weight, decreased serum TNF-alpha levels, increased apoptotic tumor cells, enhanced expression of p53 molecule in tumor tissue, and modulated tumor cell apoptotic pathway	[95]	—

2	Curcumin	Oral curcumin 8 g/day	Gemcitabine-based chemotherapy	A preliminary study in 17 patients with advanced pancreatic cancer	5 patients (29%) discontinued curcumin within few weeks due to abdominal fullness or pain (observed in 7 patients), and the dose of curcumin was reduced to 4000 mg/day. One of 11 evaluable patients (9%) had partial response, 4 (36%) had stable disease, and 6 (55%) had tumor progression	[295]	[294]

2	Curcumin	Oral curcumin 8 g/day	Gemcitabine-based chemotherapy	A phase I study in 21 gemcitabine-resistant patients with pancreatic cancer	No patients were withdrawn from this study because of the intolerability of curcumin	[96]	[294]

2	Curcumin	Curcumin was orally given from 500 mg/d for seven consecutive d by cycle (from d-4 to d + 2) and escalated until a dose-limiting toxicity should occur	Docetaxel chemotherapy	An open-label phase I trial in 14 patients with advanced and metastatic breast cancer	Maximal-tolerated dose of curcumin was established to 8000 mg/d. Some improvements as biological and clinical responses were observed in most patients	[296]	[297]

2	Curcumin	Patients received 8 g curcumin by mouth daily until disease progression, with restaging every 2 months	Individuals who underwent radiotherapy or chemotherapy <4 weeks beforehand were excluded from the study	A nonrandomized, open-label, phase II trial of curcumin in 25 patients with histologically confirmed adenocarcinoma of the pancreas	One patient had ongoing stable disease for >18 months and another had a brief, but marked tumor regression (73%) accompanied by significant increases (4-fold to 35-fold) in serum cytokine levels (IL-6, IL-8, IL-10, and IL-1 receptor antagonists). No toxicities were observed	[298]	—

2	Curcumin	Curcuma extract in proprietary capsule form was given at doses between 440 and 2200 mg/day, containing 36–180 mg of curcumin	Patients received various previous chemotherapy or radiotherapy	A preliminary study in 15 patients with advanced colorectal cancer refractory to standard chemotherapies	Curcuma extract was administered safely to patients at doses of up to 2.2 g daily, equivalent to 180 mg of curcumin but a low bioavailability of curcumin in humans was also observed	[299]	—

3	EGCG	Dose escalation proceeded according to a standard phase I design from 40 *μ*M to 440 *μ*M	Cisplatin and etoposide	Single-arm phase I study in 24 patients with unresectable stage III non-small-cell lung cancer	Dramatic regression of esophagitis was observed in 22 of 24 patients and mean pain score was reduced	[114]	[300, 301]

3	EGCG	Double-brewed green tea at 500 ml day	Platinum/taxane	Maintenance treatment after chemotherapy in 16 women with advanced stage ovarian cancer	Only 5 of 16 women remained free of recurrence after 18 months	[302]	[303]

3	EGCG	2000 mg twice a day	—	Single-arm phase II trial in 42 patients with asymptomatic, Rai stage 0 to II chronic lymphocytic leukemia who did not meet criteria to initiate conventional chemotherapy treatment	29 patients (69%) showed decline ≥ 20% in the lymphocyte count and/or a reduction ≥ 30% in the sum of the products of all lymph node areas during the 6 months of treatment	[304]	—

3	EGCG	Green tea extract 375 mg per day	—	Single-arm study in 19 hormone-refractory prostate cancer patients	No patient had a prostate-specific antigen response (i.e., at least 50% decrease from baseline), and all 19 patients were deemed to have progressive disease within 1 to 5 months	[305]	—

3	EGCG	6 g of powdered green tea extract daily	—	Phase II trial of green tea in the treatment of 42 patients with androgen-independent metastatic prostate carcinoma	Limited antineoplastic activity, as defined by a decline in PSA levels (decline > 50% in the baseline PSA value, occurred in a single patient) and 4 episodes of toxicity, was observed	[306]	—

4	Quercetin	Quercetin 20 mg orally 3 times a day in combination with curcumin (480 mg)	—	A phase I trial in patients with familial adenomatous polyposis with prior colectomy	All 5 patients had a decreased polyp number and size from baseline after a mean of 6 months of treatment with curcumin and quercetin	[307]	—

4	Quercetin	Short i.v. infusion at escalating doses from (1400 mg/m2 was recommended)	Previous chemotherapy was reported for 40/51 patients	A phase I and phase II trial in patients with various cancer no longer amenable to standard therapies	In 9 of 11 patients, lymphocyte protein tyrosine phosphorylation was inhibited. In one patient with ovarian cancer and in another patient with hepatoma, circulating tumor markers were decreased	[132]	

5	Genistein	In escalating doses (400 mg–1600 mg daily) of a multicomponent crystalline form	Concomitant gemcitabine treatment (1000 mg/m^2^)	A phase I study in 16 patients with inoperable pancreatic carcinoma	No signs of toxicity observed. The median overall survival time was 4.9 months (range 1.5–19.5 months)	[308]	—

5	Genistein	30 mg genistein or placebo capsules daily for 3–6 weeks before radical prostatectomy	—	A phase II placebo-controlled, randomized, double-blind clinical trial was conducted in 47 patients with prostate cancer	No significant effects on proliferation-, cell cycle-, apoptosis- or neuroendocrine biomarkers. Modulation of the expression of some biomarkers related to prediction and progression	([309]; [310])	

5	Genistein	Oral genistein (300 or 600 mg/d as the purified soy extract G-2535) for 14 to 21 days before surgery	—	A phase II randomized, placebo-controlled trial in 59 subjects diagnosed with urothelial bladder cancer	Reduction in bladder cancer tissue p-EGFR staining at dose of 300 mg. No other significant differences in the multitude of clinical molecular parameters measured		
6	Resveratrol	Micronized resveratrol at 5 g daily	Bortezomid	A phase II study of SRT501 (resveratrol) with bortezomib in 24 patients with relapsed and or refractory multiple myeloma	Unacceptable safety profile and minimal efficacy in patients with relapsed/refractory multiple myeloma	[170]	[311]

6	Resveratrol	Micronized resveratrol at 5 g daily	—	Phase I randomized, double-blind pilot in patients with colorectal cancer and hepatic metastases	Administration was safe. A small but significant increase in cleaved caspase-3 immunoreactivity in tumor tissue compared to placebo was detected	[171]	—

7	Silybin	Dose escalation from 2 to 12 g per day with silybin-phosphatidylcholine	—	Phase I dose-preliminary study in 3 patients with advanced hepatocellular carcinoma not eligible for other therapies	All patients died soon after enrolment and were not possible to conclude about the effects	[182]	—

7	Silybin	Dose escalation from 2.5 to 20 g per day with silybin-phytosome	—	Phase I pharmacokinetic study in 13 patients (over 70 years) with prostate cancer	No response on prostate-specific antigen was observed and one patient displayed grade 3 toxicity	[312]	—

8	PEITC	10 mg in 1 ml of olive oil, 4 times per day, for 1 week	—	Not an adjuvant therapy, but a clinical trial with a crossover design versus placebo in 18 smokers	Metabolic activation of one carcinogen was reduced by treatment	[192]	

9	Sulforaphane	200 *μ*moles/day of sulforaphane-rich extracts for a maximum period of 20 weeks	—	Phase II study in 20 patients with recurrent prostate cancer	Treatment did not lead to ≥50% PSA declines in the majority of patients. A significant lengthening of the on-treatment PSA doubling time was observed compared with the pretreatment PSA doubling time. Administration was safe with no grade 3 adverse events	[208]	—

10	Triptolide	2 weekly infusions for 3 weeks with 2 mg of a semisynthetic derivate of triptolide, which is converted to triptolide in vivo (F60008)		Phase I trial in 20 patients with advanced solid malignancy for whom standard therapy options did not exist	Treatment-induced mild grade 1-2 anaemia, fatigue, nausea, vomiting, diarrhea, and constipation. Two lethal events were observed	[219]	

11	Allicin	Local application of allicin, via gastroscopy (48 h before surgical intervention)	—	Trial on 40 patients with progressive gastric carcinoma versus 40 controls	In cancer tissues removed by surgery, cell apoptosis rate was 9.60 versus 2.20 in the control group. There were additional differences in the expression of proapoptotic genes and in cell cycle progression	[313]	

12	Berberine	20 mg kg− 1 once a day	Radiation therapy	Two arm study in 90 patients with non-small-cell lung cancer. The trial group received radiation therapy plus berberine, and the control group received radiation therapy plus a placebo for 6 weeks	Reduced the incidence of radiation-induced lung injury and improved pulmonary function	[243]	[292]

13	Piperlongumine	No clinical trial	—	—	—	—	—

14	Fisetin	No clinical trial	—	—	—	—	—

15	Phloretin	No clinical trial	—	—	—	—	—

^∗^When the natural bioactive compound was used as adjuvant therapy, otherwise not applicable. ^∗∗^Explicative references that identify mechanisms related to senescence induction of the chemotherapy drugs or treatments reported in the adjuvant therapy.
